# Role of *In Vitro* Stimulation with Lipopolysaccharide on T-Cell Activation in HIV-Infected Antiretroviral-Treated Patients

**DOI:** 10.1155/2012/935425

**Published:** 2012-02-15

**Authors:** Camilla Tincati, Giusi M. Bellistrì, Giuseppe Ancona, Esther Merlini, Antonella d'Arminio Monforte, Giulia Marchetti

**Affiliations:** Department of Medicine, Surgery and Dentistry, Clinic of Infectious Diseases and Tropical Medicine, “San Paolo” Hospital, University of Milan, Via A. di Rudinì 8, 20142 Milan, Italy

## Abstract

We investigated the effect of LPS *in vitro* stimulation on T-cell activation in HIV-infected patients with different CD4+ recovery on HAART. PBMCs from 30 HIV-positive, HAART-treated, aviremic individuals with different CD4+ reconstitution (Low Responders: CD4+ < 350/*μ*L; Intermediate Responders: CD4+ 350–599/*μ*L; High Responders: CD4+ ≥ 600/*μ*L) were cultured with LPS and the proportion of HLA-DR/CD38- and Ki67-expressing CD4+/CD8+ T-cells was measured (flow cytometry). Upon LPS stimulation, significantly higher CD4+ and CD8+HLA-DR+ cells were shown in LR and IR versus HIV-negative controls. While no differences in the proportion of LPS-stimulated CD4+CD38+ cells were recorded amongst HIV-positive subgroups, CD8+CD38+ cells were more elevated in patients with lower CD4+ recovery on HAART (i.e., LR and IR). Upon *in vitro* LPS stimulation, HLA-DR and CD38 expression on T-cells are differentially regulated. While HLA-DR induction reflects impaired CD4+ reconstitution on HAART, cell-surface CD38 expression is increased only on CD8+ T-cells, allowing to speculate that the sole induction of CD38 on CD4+ cells may not be sufficient to depict LPS-driven immune activation in HIV.

## 1. Introduction

Untreated HIV disease is characterized by high levels of T-cell activation which account for the progressive depletion of CD4+ T-cells [1, 2].

Microbial translocation, which occurs following the breakdown of the gastrointestinal barrier, has been put forward as a possible mechanism underlying immune activation in HIV disease [2, 3]. Most interestingly, microbial translocation-induced T-cell hyperactivation may also represent a cause of impaired immune restoration on virologically suppressive HAART [4–6], as suggested by high levels of circulating lipopolysaccharide (LPS) in patients with poor CD4+ T-cell recovery in course of effective treatment [4].

In keeping with these observations, *in vitro* exposure of PBMCs to LPS and other Toll-Like Receptor (TLR) agonists has been shown to induce the expression of CD38, indicator of T-cell activation, on CD4+ and CD8+ T-cells in healthy individuals [7], thus suggesting a model for HIV pathogenesis. Indeed, CD38 upregulation on CD8+ cells has been consistently described in HIV infection and correlates with disease progression better than HIV RNA load [8–11]; conversely, the issue of whether high CD38 expression on CD4+ T-cells is a marker of poor prognosis [8, 9, 11, 12] has been debated for some time [13]. Taken together, these findings imply that the actual biological significance of CD38 in the context of HIV/AIDS remains elusive, given the multiple functions it retains [14]. Indeed, along with its association with T-cell activation, recent data have demonstrated that CD38 on CD4+ cells may also identify a hypoproliferating cell subset, thus offsetting the paradigm of CD38 as T-cell activation marker [13, 15].

In the light of these premises, we investigated the dynamics of HLA-DR and CD38 expression on peripheral T-cells following selective *in vitro* LPS stimulation of PBMCs from HIV-infected HAART-treated individuals. We hypothesized that LPS may account for diverse levels of immune activation in HIV-infected patients according to the degree of immunological recovery in course of suppressive HAART. We also hypothesized that HLA-DR and CD38 may not be equivalent in reflecting the extent of T-cell activation following *in vitro* stimulation with LPS in the setting of HIV disease.

## 2. Materials and Methods

### 2.1. Patients

HIV-positive, HAART-treated patients for at least 12 months, with undetectable HIV RNA load (<40 cp/mL) and a CD4+ T-cell nadir <250/*μ*L, were consecutively enrolled at the Clinic of Infectious Diseases and Tropical Medicine, “San Paolo” Hospital, University of Milan, Italy. Patients were divided into 3 groups according to the degree of immune-reconstitution in course of HAART: high responders (HRs) with CD4+ ≥600/*μ*L, and low responders (LRs) with CD4+ <350/*μ*L; intermediate responders (IR) with CD4+ 350–599/*μ*L. As controls, HIV-negative age-matched subjects were studied. Written informed consent forms approved by the Ethical Committee of the “San Paolo” Hospital, University of Milan, Italy, were obtained from all participants.

### 2.2. Laboratory Methods

#### 2.2.1. Plasma HIV RNA Levels

Plasma HIV-1 RNA levels were quantified by means of a nucleic acid signal amplification assay (Abbott Real-Time PCR assay), which has a lower detection limit of 40 HIV RNA copies/mL of plasma.

#### 2.2.2. Lymphocyte Immunophenotype Analysis

Fresh peripheral blood was drawn from all study participants in EDTA-containing tubes and PBMCs were separated by Ficoll-Histopaque technique (Biocoll separating solution, BIOSPA) (T0). Cells were counted and 5×10^6^ cells were cultured in R10 medium alone (composition per 100 mL R10: 88 mL RPMI, 10 mL fetal bovine serum, 1 mL [100 UI/mL] L-glutamine, and 1 mL [100 UI/mL] penicillin/streptomycin; Euroclone, Italy) (unstimulated, US) or in medium supplemented with LPS for 24 hours (T1) and 48 hours (T2) (E. coli; 026 : B6*C, Sigma-Aldrich, Milan, Italy, 20 ng/mL; stimulated, STIM). Prior to and following stimulation, cells were recounted and stained (CD4+/CD8-PerCP Cy5.5, HLA-DR-FITC, CD38-PE; Ki67-FITC, Becton Dickinson, San Josè, CA, USA) for flow cytometric analysis of HLA-DR, CD38, and Ki67 expression on T-cells (Cytomics FC500, Beckman Coulter, Hialeah, FL, USA) using CXP 2.2. software.

### 2.3. Statistics

Data were analyzed with GraphPad 5 PRISM software. Mann-Whitney *U*-test, Kruskall-Wallis and Wilcoxon tests were used for statistics. All statistical tests were 2-sided and differences were considered statistically significant at *P* < 0.05. 

## 3. Results

### 3.1. Patients

30 HIV-positive, HAART-treated patients with undetectable HIV RNA load were enrolled. According to the degree of immune-reconstitution on HAART, 9 HIV-positive patients were HR (CD4+ ≥600/*μ*L), 9 resulted LR (CD4+ <350/*μ*L), and 12 intermediate IR CD4+ 350–599/*μ*L. 15 HIV-negative age-matched subjects were also studied.

Duration of HIV infection was comparable in all subjects (LR: 7 months, IQR 5–19; IR: 13 months, IQR 8–22; HR: 12 months, IQR 6–23; *P* > 0.05 for all comparisons; Table 1). All patients were on HAART for at least 12 months prior to evaluation, with no differences in terms of HAART duration (LR: 71 months, IQR 46–141; IR: 106 months, IQR 65–149; HR 71 months, IQR 39–132; *P* > 0.05 for all comparisons; Table 1) and drug regimen (Table 1).

HRs were significantly younger compared to LRs (HR: 39 years, IQR 35–44; LR: 50 years, IQR 45–56; *P* = 0.02; Table 1) and presented higher absolute CD4+ T-cell counts as defined by inclusion criteria (LR: 260/*μ*L, IQR 215–339; IR: 498/*μ*L, IQR 425–537; HR: 726/*μ*L, IQR 608–863; *P* < 0.01 for all comparisons; Table 1). A trend to lower CD4+ T-cell nadir was found in patients with less efficient immune-reconstitution, (LR: 90/*μ*L, IQR 51–179; IR: 105/*μ*L, IQR 45–208; HR: 204/*μ*L, IQR 56–240; *P* > 0.05 for all comparisons) reaching statistical significance for percentage values in LR versus HR (LR: 8%, IQR 5–17; HR: 26%, IQR 22–30; *P* = 0.03; Table 1).

No other significant differences in demographic and HIV-related parameters were observed among groups (Table 1).

### 3.2. Lymphocyte Immunophenotype Analysis

#### 3.2.1. HLA-DR Expression on CD4+ and CD8+ T-Cells upon LPS Stimulation

**Figure 1:**
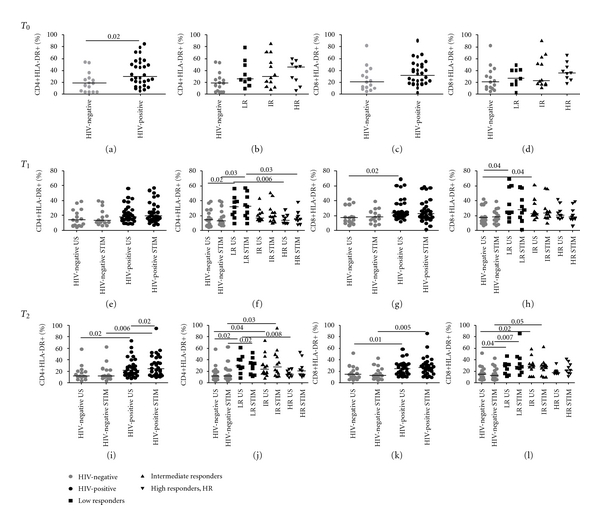
HLA-DR-expressing CD4+/CD8+ T-cells in HIV-negative and HIV-positive patients prior to and following LPS stimulation. HLA-DR expression was measured on freshly ficoll-separated CD4+ and CD8+ T-cells at baseline (T0, *top*), and following 24- (T1, *middle*) and 48-hour (T2, *bottom*) LPS stimulation. PBMCs were cultured in medium alone (unstimulated, US) or in medium with 20 ng/mL LPS (stimulated, STIM). At T0, HIV-positive individuals displayed higher HLA-DR+CD4+ (*P* = 0.02, (a)) and HLA-DR+CD8+ proportions (c), with no differences amongst HIV-positive subgroups ((b) and (d)). At T1, HIV-positive patients maintained increased HLA-DR+CD4+ (e) and CD8+ cells (g); significantly higher HLA-DR+CD4+ levels were detected in LR versus HR and negative controls (*P* = 0.03 for both comparisons, (f)). At T2, HIV-infected individuals displayed significantly higher HLA-DR+CD4+ (*P* = 0.006, (i)) and CD8+ cells compared to controls (*P* = 0.005, (k)). Following stimulation, significantly greater proportions of HLA-DR-expressing CD4+ were measured in LR (*P* = 0.02, (j)) and IR (*P* = 0.03, (j)) compared to controls. Comparable results were detected in the CD8+ compartment upon LPS stimulation (LR versus HIV-negative, *P* = 0.007, (l); IR versus HIV-negative, *P* = 0.05, (l)). *P* values in the results section refer to stimulated samples only.

At T0, HIV-positive patients displayed a tendency to higher HLA-DR+CD4+/CD8+ compared to controls, reaching significance for the CD4+ T-cell subpopulation (CD4+: 30%, IQR 20–54 versus 19%, IQR 5–28; *P* = 0.02; Figure 1(a); CD8+: 32%, IQR 18–50 versus 21% IQR 11–39; *P* = 0.07; Figure 1(c)), with no differences according to immune-reconstitution (Figures 1(b), and 1(d)).

At T1, following LPS stimulation, HIV-infected individuals as a whole displayed a non-significant trend to increased HLA-DR+CD4+ (19%, IQR 14–33 versus 13%, IQR 11–26; *P* = 0.08; Figure 1(e)) and HLA-DR+CD8+ (23%, IQR 17–37 versus 18%, IQR 13–29 *P* = 0.2; Figure 1(g)) when compared to healthy controls. Interestingly, when analysing patients according to CD4+ recovery on HAART, LPS stimulation resulted in significant upregulation of HLA-DR on CD4+ T-cells in LR (32%, IQR 19–50) compared to HR (15%, IQR 11–21; *P* = 0.03; Figure 1(f)) and controls (*P* = 0.03; Figure 1(f)). Conversely, no differences in HLA-DR-expressing proportions upon LPS stimulation were observed in the CD8+ compartment at this timepoint (LR: 28%, IQR 16–50; IR: 24%, IQR 18–50; HR: 18%, IQR 15–32; *P* > 0.05 for all comparisons; Figure 1(h)).

At T2, HIV-positive subjects displayed significantly higher LPS-induced HLA-DR+CD4+ (24%, IQR 14–44.3 versus 12%, IQR 8–23; *P* = 0.006; (i)) and HLA-DR+CD8+ cells (26%, IQR 18–34 versus 13%, IQR 7–25; *P* = 0.005; (k)). Following stimulation with LPS, a CD4+ T-cell activation hierarchy was maintained in LR (32%, IQR 15–40) and IR (28%, 14–48) over HR (21%, IQR 13–37; *P* = 0.4; *P* = 0.5, resp.; (j)), reaching statistical significance in comparison with HIV-negative controls (*P* = 0.02 and *P* = 0.03, resp.; (j)). Similar results were observed in terms of LPS-induced expression of HLA-DR on CD8+ cells, with increased levels in LR (28%, IQR 22–45) and IR (28%, IQR 15–33; (l)) compared to controls (*P* = 0.007 and *P* = 0.05) (l). 

A graphical time-course representation of the effect of LPS stimulation on HLA-DR in HIV-infected individuals with different response to HAART and in HIV-negative controls is summarized in Figure 3. 

#### 3.2.2. CD38 Expression on CD4+ and CD8+ T-Cells upon LPS Stimulation

**Figure 2:**
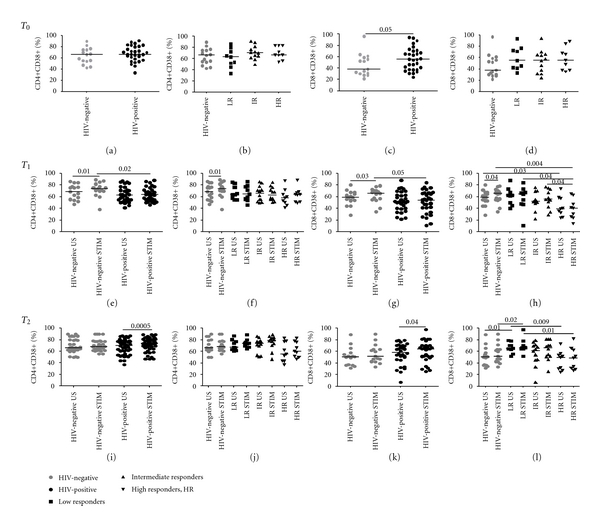
CD38-expressing CD4+/CD8+ T-cells in HIV-negative and HIV-positive patients prior to and following LPS stimulation. CD38 expression was measured on freshly ficoll-separated CD4+ and CD8+ T-cells at baseline (T0, *top*) and following 24- (T1, *middle*) and 48-hour (T2, *bottom*) LPS stimulation. PBMCs were cultured in medium alone (unstimulated, US) or in medium with 20 ng/mL LPS (stimulated, STIM). At T0, comparable CD4+CD38+ were detected in HIV-positive patients and controls (a); however, the former displayed markedly higher CD8+CD38+ (*P* = 0.05, (c)), with no differences amongst HIV-positive subgroups. At T1, a significantly lower expression of CD38 was detected on CD4+ (*P* = 0.02, (e)) and CD8+ cells (*P* = 0.05, (g)) from HIV-positive individuals despite LPS stimulation. While no differences in CD4+CD38+ were recorded amongst HIV-positive subgroups (f), CD8+CD38+ cells were significantly higher in LR and IR compared to HR (*P* = 0.04 for both comparisons, (h). At T2, LR presented highest CD38+CD8+ compared to HR (*P* = 0.01, (l)) and controls (*P* = 0.02, (l)) with no differences in CD4+CD38+ (j). *P* values in the results section refer to stimulated samples only.

Baseline (T0) CD38+CD4+ cells were comparable in HIV-positive and HIV-negative patients (66%, IQR 58–80 versus 66%, IQR 54–75, *P* = 0.4; Figure 2(a)). Conversely, CD38+CD8+ cells were higher in HIV-positive patients (55%, IQR 38–71 versus 38%, IQR 30–59, *P* = 0.05; Figure 2(c)). In both cases, no differences amongst HIV-infected subgroups were detected (Figures 2(b), and 2(d)).

Most interestingly, cells from HIV-infected patients failed to respond to LPS stimulation at T1, displaying significantly lower proportions of CD38+CD4+ (64%, IQR 55–72.7 versus 73%, IQR 70–82; *P* = 0.02; Figure 2(e)) and CD38+CD8+ (54%, IQR 39–67 versus 65%, IQR 55–70, *P* = 0.05; Figure 2(g)) compared to HIV-negative controls. However, within HIV-infected subjects, LR and IR showed higher CD38+CD8+ (65%, IQR 48–70 and 56%, IQR 45–71, resp.) compared to HR upon LPS stimulation (41%, IQR 25–55; *P* = 0.04 for both comparisons; Figure 2(h)), while CD38+CD4+ cells did not vary according to immune-reconstitution (LR: 64%, IQR 55–78; IR: 62%, IQR 55–75; HR: 64%, IQR 51–67; *P* > 0.05 for all comparisons; Figure 2(f)).

At T2, HIV-positive and healthy individuals displayed comparable CD38+CD4+ and CD8+ following LPS stimulation (74%, IQR 65–80 versus 66%, IQR 61–77; *P* = 0.3; Figure 2(i); 64%, IQR 50–70 versus 52%, IQR 42–63; *P* = 0.2; Figure 2(k)). similar to T1 data, LR presented higher CD38+CD8+ than HR (66%, IQR 64–74 versus 49%, IQR 30–64, *P* = 0.01Figure 2(l)) and controls (*P* = 0.02; Figure 2(l)), whereas no differences in CD38+CD4+ cells were noted amongst HIV-positive subgroups (LR: 74%, IQR 68–78; IR: 79%, IQR 71–83; HR: 60%, IQR 50–77; *P* > 0.05 for all comparisons; Figure 2(j)).

**Figure 3:**
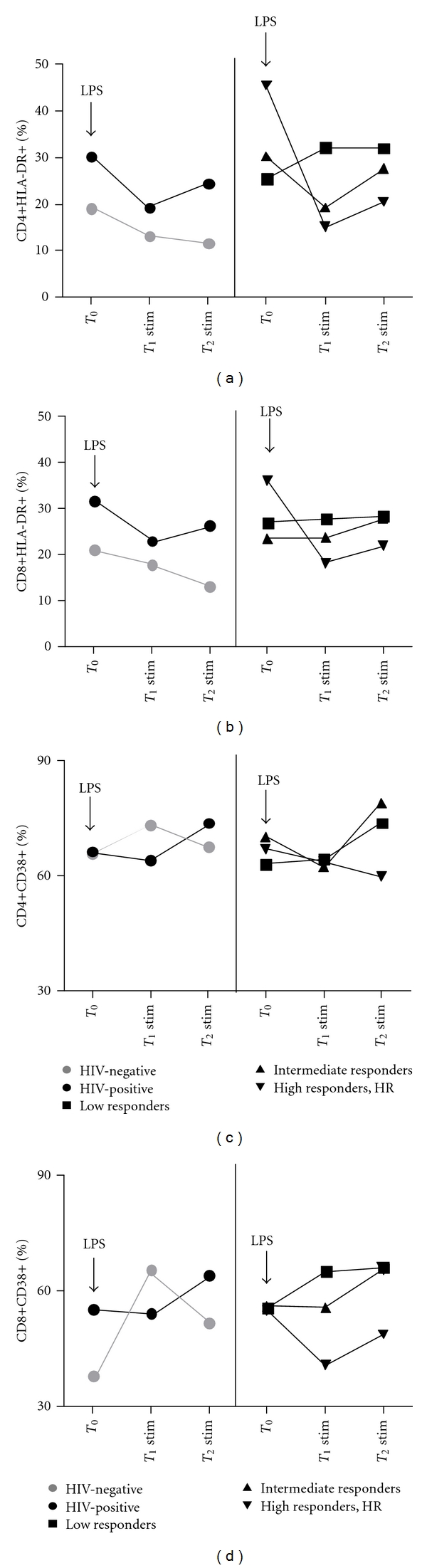
Time course representation of the effect of LPS on HLA-DR and CD38 expression on CD4+ and CD8+ T-cells. Median HLA-DR ((a) and (b)) and CD38 ((c) and (d)) expression on CD4+ and CD8+ T-cells in HIV-positive and HIV-negative individuals (left portion of graphs) and in HIV-infected subjects with different immunological outcome in course of HAART (right portion of graphs) at baseline (T0) and following LPS stimulation (T1 stim, T2 stim).

A graphical time-course representation of the effect of LPS stimulation on CD38 in HIV-infected individuals with different response to HAART and in HIV-negative controls is summarized in Figure 3. 

#### 3.2.3. HLA- DR/CD38 Co-expression on CD4+ and CD8+ T-Cells upon LPS Stimulation

At T0 comparable HLA-DR+CD38+CD4+ cells were observed in HIV-negative (15.4%, IQR 2.1–25.9) and HIV-positive patients (23.5%, IQR 7.2–48.8; *P* = 0.08) with no differences among HIV subgroups (LR: 20.2%, IQR 6.9–42; IR: 24.5%, IQR 8.1–63.1; HR: 41.5%, IQR 12.1–49.5; *P* > 0.05 for all comparisons).

At T1, LPS did not account for significant changes in HLA-DR/CD38 coexpression on CD4+ T-cells compared to baseline values.

A completely different scenario was pictured upon LPS stimulation at T2, with significantly higher HLADR+CD38+CD4+ in HIV-positive patients as a whole (20.3%, IQR 12.1–39.4) and in LR subjects (LR: 20.3%, IQR 12.8–34.4) compared to controls (9.5%, IQR 6.6–19.0; *P* = 0.04 and *P* = 0.03, resp.).

Similar results were observed in HLA-DR/CD38 coexpression on CD8+ T-cells, given that no differences among study groups were detected at T0: HIV-negative: 13.6%, IQR 4.0–24.9; HIV-positive: 18.3%, IQR 12.1–42.2; LR: 17.9% IQR 12.2–28.7; IR: 14.1%, IQR 8.0–45.7; HR: 30.5%, IQR 12.8–46.7 (*P* > 0.05 for all comparisons).

 While LPS stimulation did not lead to significant differences in terms of HLA-DR/CD38 induction on CD8+ cells at T1, significantly higher HLADR+CD38+CD8+ cells were detected in HIV-positive patients compared to controls (20.5%, IQR 10.5–29.0 versus 8.7%, IQR 4.9–20.1; *P* = 0.02) at T2. 

When examining patients with different degree of immune-reconstitution, LR maintained activation CD8+ activation hierarchy over HIV-negative individuals, with significantly more HLADR+CD38+CD8+ cells (LR: 24%, IQR 14.6–36.4; **P** = 0.02).

#### 3.2.4. Ki67 Expression on CD4+ and CD8+ T-Cells upon LPS Stimulation

**Figure 4:**
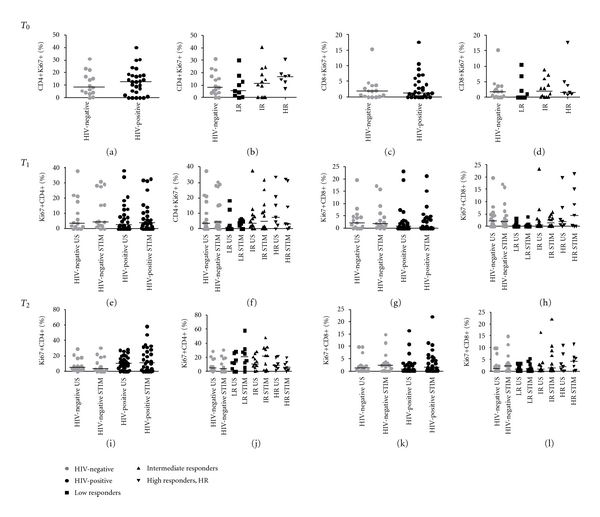
Ki67-expressing CD4+/CD8+ T-cells in HIV-negative and HIV-positive patients prior to and following LPS stimulation. Ki67 expression was measured on freshly ficoll-separated CD4+ and CD8+ T-cells at baseline (T0, *top*) and following 24- (T1, *middle*) and 48-hour (T2, *bottom*) LPS stimulation. PBMCs were cultured in medium alone (unstimulated, US) or in medium with 20 ng/mL LPS (stimulated, STIM). At T0, no differences between HIV-negative controls and HIV-positive subjects were measured in Ki67 expression on CD4+ (a) and CD8+ T-cells (c). T-cell proliferation levels did not vary according to the degree of immune-reconstitution in course of HAART ((b) and(d)). At T1, LPS stimulation did not account for significant increases in cell proliferation ((e)–(h)). At T2, a trend to increased Ki67 levels on CD4+ T-cells upon stimulation was detected in LR compared to HR and negative controls (j); no differences were detected in terms of Ki67+CD8+ T-cells proliferation among study groups following 48-hour stimulation with LPS ((k) and (l)).

At T0, no differences between HIV-negative controls and HIV-positive subjects were measured in Ki67 expression on CD4+ (8.2%, IQR 4.0–17.2 versus 12.7%, IQR 2.6–18.2, res.; **P** = 0.7; Figure 4(a)) and CD8+ T-cells (1.9%, IQR 0.1–3.7 versus 1.25; IQR 0.0–4.8; **P** = 0.8; Figure 4(c)). T-cell proliferation levels did not vary according to the degree of immune-reconstitution in course of HAART, despite trend to higher Ki67 expression on CD4+ T-cells in HR (17%, IQR 12.6–18.9) compared to LR (5.5%, IQR 1.4–15.5; **P** = 0.07; Figure 4(b)).

At T1, LPS stimulation did not account for significant increases in cell proliferation (Figures 4(e)–4(h)). Interestingly, however at T2, a trend to increased Ki67 levels on CD4+ T-cells upon stimulation was detected in LR (20.5%, IQR 7.5–31.0) compared to HR (5.5%, IQR 2.0–12.6; **P** = 0.08) and negative controls (3.9%, IQR 0.4–18.7; **P** = 0.08; Figure 4(j)), thus mirroring the state of CD4+ T-cell activation through the expression of HLA-DR (Figure 1(j)).

No differences were detected in terms of CD8+ T-cell proliferation among study groups following 48-hour stimulation with LPS (Figures 4(k), and 4(l)). 

## 4. Discussion

We hereby investigated the induction of CD38, HLA-DR and Ki67 on CD4+ and CD8+ T-cells following selective *in vitro* LPS stimulation in HIV-infected individuals with different immunological recovery upon virologically suppressive HAART.

We enrolled patients on stable antiretroviral therapy and with comparable demographic and viro-immunological parameters. Of note, patients with less efficient immune-reconstitution were significantly older and presented decreased T CD4+ cell nadir compared to subjects displaying sustained CD4+ T-cell recovery; this finding is in keeping with literature data demonstrating that age and CD4+ cell counts at HAART initiation are determinants of incomplete immunological reconstitution in course of suppressive HAART [16].

Our data show a differential effect of LPS *in vitro* stimulation on the cell-surface expression of two T-cell activation markers, that is, HLA-DR and CD38: (i) despite an overall reduction of HLA-DR+ T-cells, patients with an inefficient response to HAART display a higher proportion of activated HLA-DR+CD4+/CD8+ compared to individuals with better CD4+ recovery; (ii) CD38 expression is induced on T-cells, and yet only the CD38+CD8+ pool is significantly expanded according to the degree of immunological impairment.

HIV infection is characterized by high levels of immune activation [1, 2] which is a major cause of the progressive CD4+ T-cell loss in untreated disease [1, 2, 8–10] and impaired immunological recovery in course of HAART [5, 17]. A possible driver of immune activation is the translocation of bacterial bioproducts, mainly LPS, through the gastrointestinal barrier to the systemic circulation [2, 3]. Indeed, literature findings show that non-progressive disease in SIV natural host primates associates with no microbial translocation and/or immune activation [18, 19]; conversely, microbial translocation has been indirectly implicated in driving immune activation in chronically HIV-infected humans and SIV-infected Rhesus macaques [19]. In keeping with this model, in HIV-negative individuals, PBMCs stimulated* in vitro* with LPS have been demonstrated to increase the expression of CD38, and not HLA-DR [7].

Consistent with these findings and with recent data showing a dramatic decline in the percentages of DR+ subsets following PBMCs *in vitro* cultures [20], we hereby show a reduction of HLA-DR+CD4+/CD8+ cells following LPS *in vitro* stimulation in both healthy donors and HIV-infected individuals. Interestingly enough however, when evaluating HIV-patients according to the degree of immune recovery on HAART, while HLA-DR expression was reduced in patients with good CD4+ recovery, subjects with lower CD4+ reconstitution, (i.e., LR and IR) maintained stable HLA-DR-expressing T-cells. The net outcome of such diverse effect of LPS stimulation according to the extent of CD4+ recovery, is that subjects with poor immunological recovery display significantly higher HLA-DR+ T-cell proportions. Of note, LPS accounted for a higher proportion of proliferating Ki67+ T-cells in the CD4+ subset alone, thus suggesting that the combined effect of microbial stimulation on T-cell proliferation and activation seems to be restricted to the CD4+ cell compartment.

By demonstrating highest LPS-dependant HLA-DR+ T-cells exclusively in patients with lowest CD4+ recovery, our findings add up to the consolidated bulk of evidence on the inverse correlation between HLA-DR expression and CD4+ lymphopenia [10, 17]. Thus, HLA-DR might be a faithful marker of T-cell activation secondary to microbial translocation in the setting of severe immune impairment.

A different trend was shown in CD38-expressing T-cells upon LPS stimulation.

The time-course analysis of the effect of LPS stimulation on CD38 expression showed an overall rise in CD38-expressing T-cells that was, however, delayed in HIV-infected patients compared to healthy controls (i.e., 48 versus 24 hours). Most interestingly, when investigating HIV-positive subjects with different immune recovery separately, only patients with inefficient CD4+ reconstitution (i.e., LR and IR) displayed an expansion of the CD38+ T-cell pool, whereas individuals with good immunological response (HR) maintained a stable expression of CD38 on T-cells overtime. Of note, although CD38 appeared selectively induced on CD4+/CD8+ cells upon longer stimulation, only CD38+CD8+ cells displayed a linear relationship with immune impairment on HAART, with LR/IR patients showing higher LPS-stimulated CD38+CD8+. Conversely, no differences in CD38+CD4+ were shown according to the degree of immune reconstitution.

These findings are consistent with *in vivo* data showing that CD38+CD8+ T-cells are an independent predictor of disease progression [8–11, 17]; although CD38 has been described to have a similar prognostic value when expressed on T CD4+ cells, this seems less consistent [8, 11, 12].

By showing higher LPS-induced expansion of CD38+CD8+ cells, our findings suggest a diverse regulation of CD38 in CD4+ and CD8+ T-cell compartments and allow to speculate that the sole expression of CD38+ on CD4+ T-cells may not be sufficient to portray the state of immune activation in HIV disease. Indeed, CD38 has been shown to be constitutively expressed on phenotypically naïve T-cells [13, 15, 21], thus offsetting its role as an activation marker in the CD4 pool. Indeed, in patients with impaired immunological recovery in course of HAART, Massanella et al. recently reported lower proportion of CD38+CD45RA+CD4+ and higher percentage of CD38+CD45RA-CD4+ cells, probably reflecting a reduced naive compartment and a higher level of activation in CD45RA- cells [21]. This finding is in keeping with data from Benito et al. [12] who showed that CD38 expression on CD4+ cells correlated with disease progression when associated with the expression of phenotypic markers of mermory/activated (CD45R0 or HLA-DR).

In our study, the co-induction of HLA-DR and CD38 on CD4+ and CD8+ T-cells followed similar kinetics upon longer (48 hours) stimulation with LPS, with increased levels of both molecules in subjects with severe immune impairment on HAART. This finding, together with the differential expression of single cell surface molecules, once again suggests that CD38 on CD4+ cells may not faithfully mirror activation in HIV disease.

The present study has several limitations. Our finding of an *in vitro* effect of LPS stimulation in reducing the expression of activation markers cannot exclude the possibility of increased cellular death following LPS challenge, as previously shown in HIV-negative patients [7].

Our research was not designed to define the mechanisms underlying variable LPS effects and is therefore descriptive in nature. Functional data on stimulated PBMCs, such as the production of type I interferons and other cytokines, would help give a qualitative answer to what activated cells produce upon microbial challenge. In addition, data on APC (macrophages, dendritic cells) activation and signalling following TLR recognition and on the cooperation between APCs and T-cells would shed light on the precise mechanisms underlying T-lymphocyte activation upon microbial stimulation.

As a further limitation, we chose to investigate the effect of LPS alone on T-cell activation. However, given that the composition of translocating microflora may influence the immunological response in course of treatment [22], it would be interesting to study the effect of other stimuli (e.g., PHA, anti-CD3, PMA) as well as other TLR ligands, representing microbial components from Gram-positive bacteria (e.g., Pam3CysK4, TLR1/2 agonist; FSL-1, TLR6/2 agonist) or viral agents (e.g., ssRNA40, TLR8 agonist; CpG oligonucleotide, TLR9 agonist) on T-cell activation and response to HAART.

Our results thus advocate further functional studies to gain deeper insight into the regulation of T-cell activation by TLR agonists in course of HIV disease and response to therapy. These data might provide the scientific background to investigate/explore alternative/adjuvant therapeutical approaches in HIV infection aimed at manipulating the negative effects of LPS in inducing immune activation [23].

## Figures and Tables

**Table 1:** Demographic characteristics and viro-immunological parameters of the patients in study.

Characteristic	LR (*n* = 9)	IR (*n* = 12)	HR (*n* = 9)
Age, years (IQR)	50 (45–56)	48 (38–60)	39 (35–44)^a^
Sex, *F* (%)	2 (22)	3 (25)	3 (33)
Risk factors for HIV infection			
MSM, *n* (%) heterosexual, *n* (%) IDU, *n* (%)	2 (22) 6 (67) 1 (11)	5 (42) 4 (33) 3 (25)	1 (11) 3 (33) 5 (56)
Duration of HIV infection, years (IQR)	7 (5–19)	13 (8–22)	12 (6–23)
HAART duration, months (IQR)	71 (46–141)	106 (65–149)	71 (39–132)
Diagnosis of AIDS, *n*	4	3	2
Absolute T CD4+ cell counts, cell/*μ*L (IQR)			
at the time of study nadir	260 (215–339) 90 (51–179)	498 (425–537) 105 (45–208)	726 (608–863)^a^ 204 (56–240)
Percentage T CD4+ cell counts, % (IQR)			
at the time of study nadir	26 (16–34) 8 (5–17)	27 (22–33) 18 (13–22)	31 (25–43) 26 (22–30)
Plasma HIV RNA, log_10_cp/mL (IQR)			
zenith at the time of study	5.3 (4.5–5.9) 1.7	5.4 (4.1–5.7) 1.7	4.4 (3.9–5.2) 1.7
HAART regimen Number of patients at the time of study			
NRTI+PI NRTI+NNRTI Other	5 2 2	7 4 1	4 3 2

Data are presented as median and interquartile range (IQR). LR: Low Responders (CD4+<350/*μ*L, HIV RNA <40 cp/mL); IR: Intermediate Responders (CD4+ 350–599/*μ*L, HIV RNA <40 cp/mL); HR: High Responders (CD4+ ≥600/*μ*L, HIV RNA <40 cp/mL); MSM: Men having sex with men; IDU: Intravenous Drug Users; HAART: highly active antiretroviral therapy; NRTI: nucleoside reverse-transcriptase inhibitor; NNRTI: nonnucleoside reverse-transcriptase inhibitor; PI: protease inhibitor. ^a^ *P* < 0.05 for differences among groups.
